# Supplementary material for: Quantification of Histone H1 Subtypes Using Targeted Proteomics
Source: Biomolecules. 2024 Sep 27;14(10):1221. doi: 10.3390/biom14101221 (PMC11506705; doi:10.3390/biom14101221)
Supplement: Supplementary file 1 [file biomolecules-14-01221-s001.zip › biomolecules-3075036-original-images.pdf]

Raw Western blot images related to Figure 3

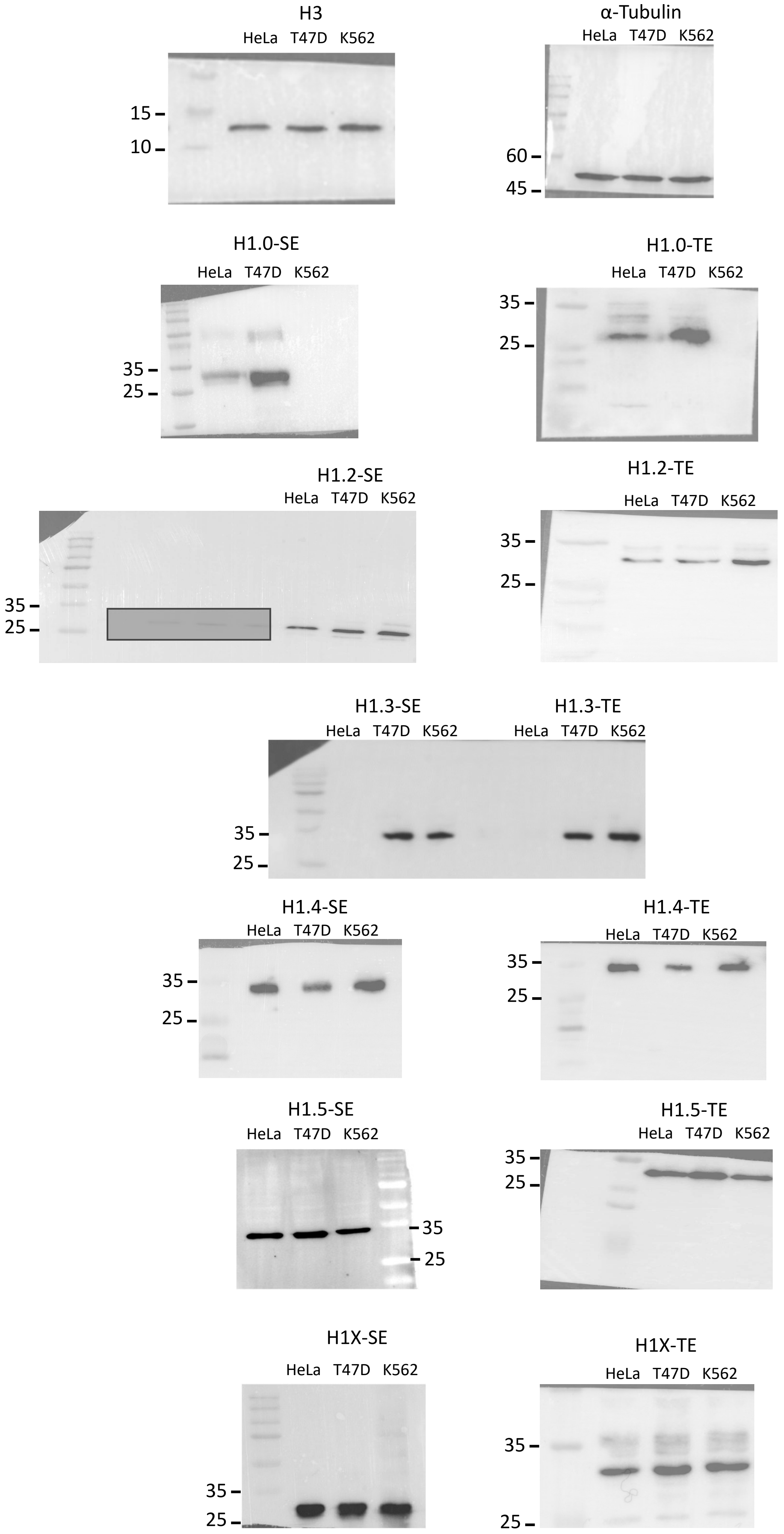

SE, sulfuric extract. TE, Total extract. Loading controls correspond to the same experiment of the rest of the images. Gray box covers bands corresponding to other experiment.
