# Supplementary material for: Quantification of Histone H1 Subtypes Using Targeted Proteomics
Source: Biomolecules. 2024 Sep 27;14(10):1221. doi: 10.3390/biom14101221 (PMC11506705; doi:10.3390/biom14101221)
Supplement: Supplementary file 1 [file biomolecules-14-01221-s001.zip › Supplementary Figures-R1.pdf]

Figure S1

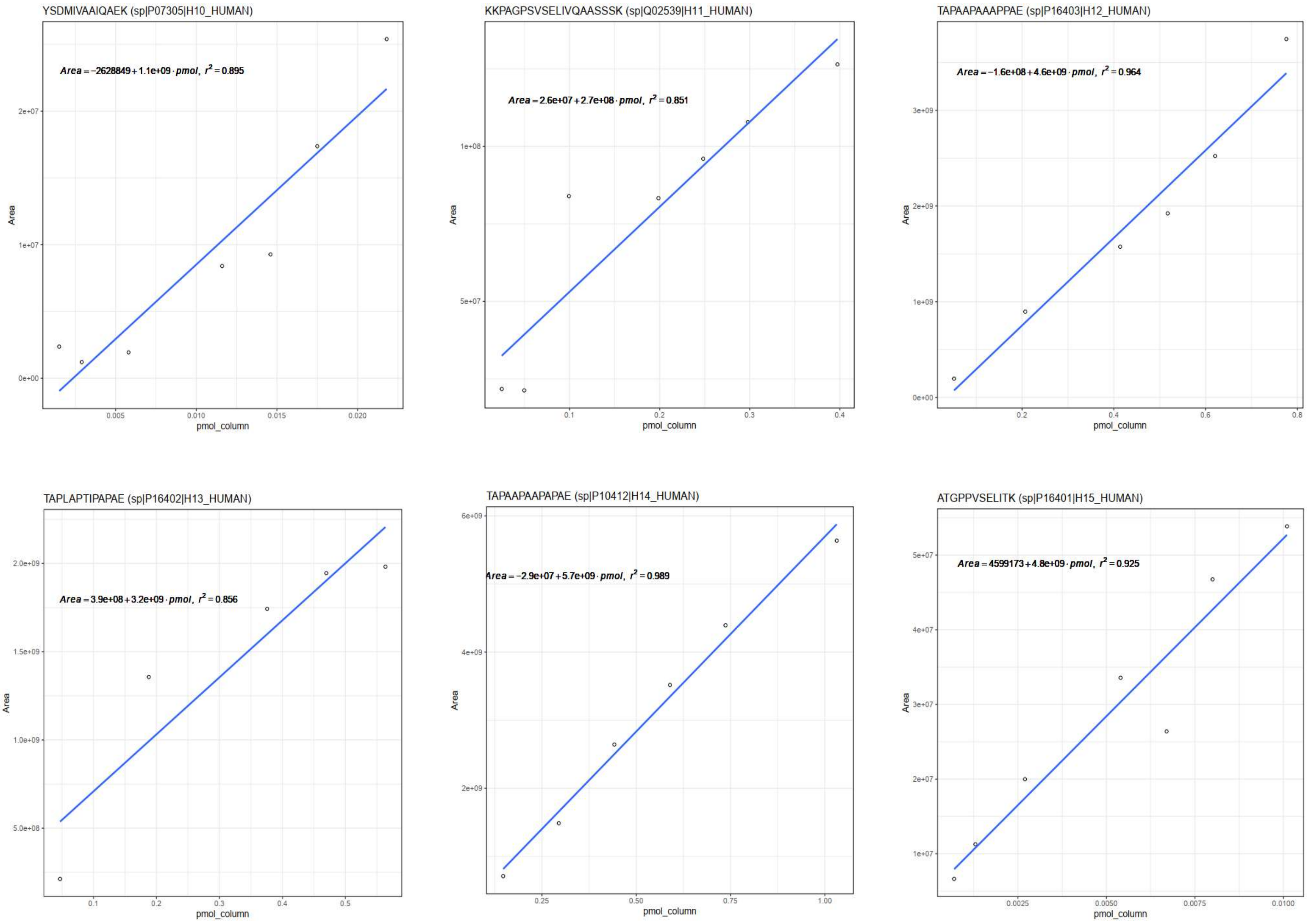

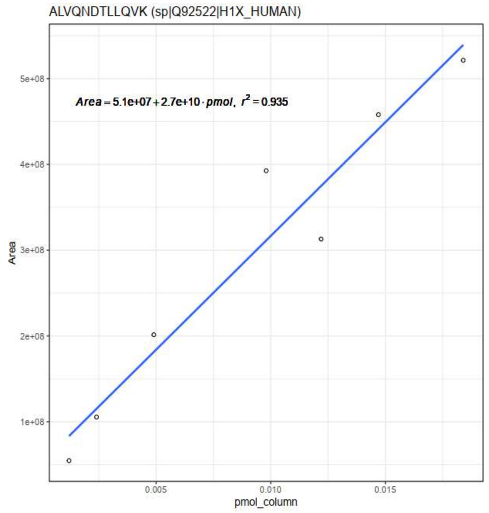

Figure S1. Initial calibration curves of the heavy peptides selected for quantification of H1 subtypes. Peptides were disolved in 3% ACN and 1% FA, as described in materials and methods.

Figure S2

A

H1.2: TAPAAPAAAPPAE; m/z 567.7931

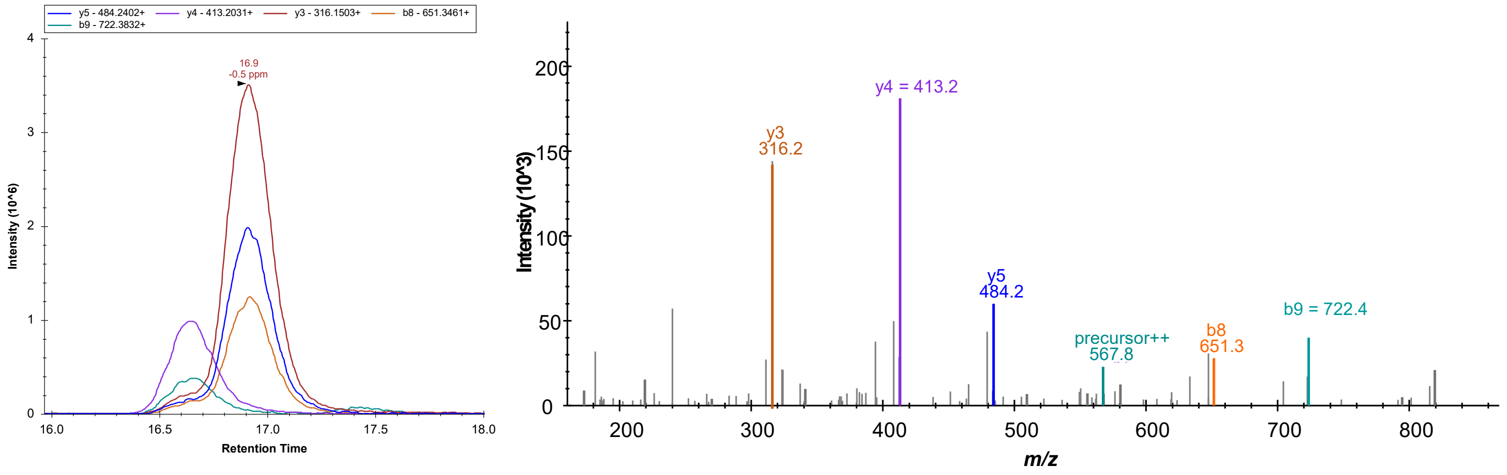

B

H1.4: TAPAAPAAPAPAE; m/z 567.7931

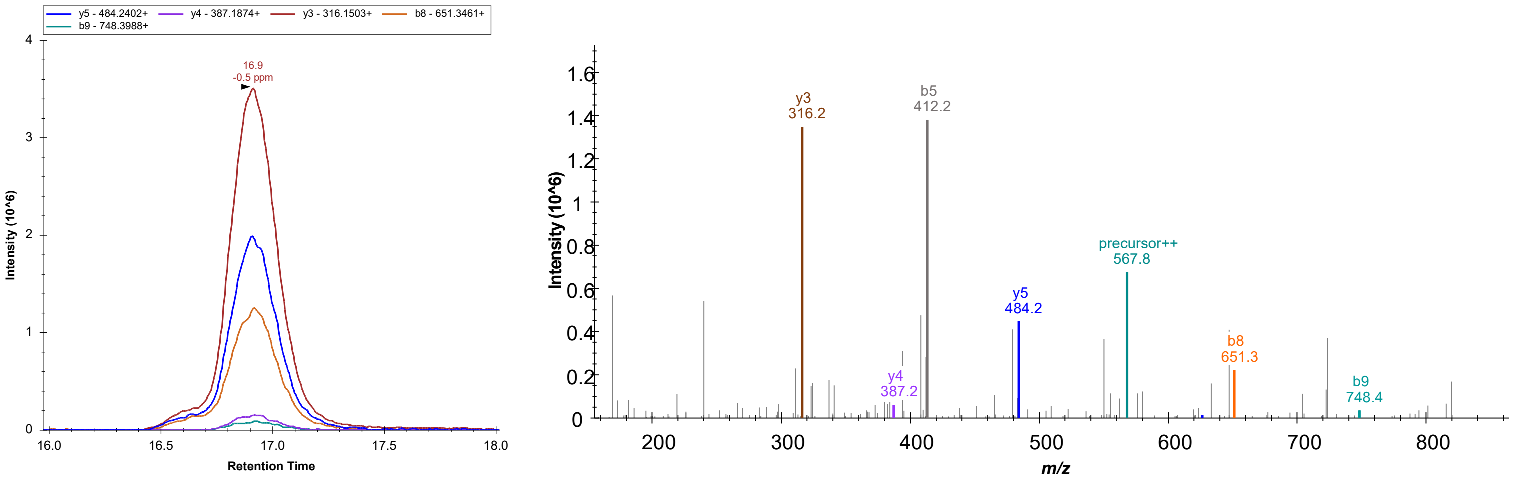

Figure S2. Analysis of H1.2 and H1.4 selected peptides. Extracted ion chromatogram of different transitions from Skyline and fragmentation spectra of, A. H1.2 selected peptide and B. H1.4 selected peptide. Only transitions y4 and b9 were used to quantify these two peptides, as they are the only transitions showing different masses.

Figure S3

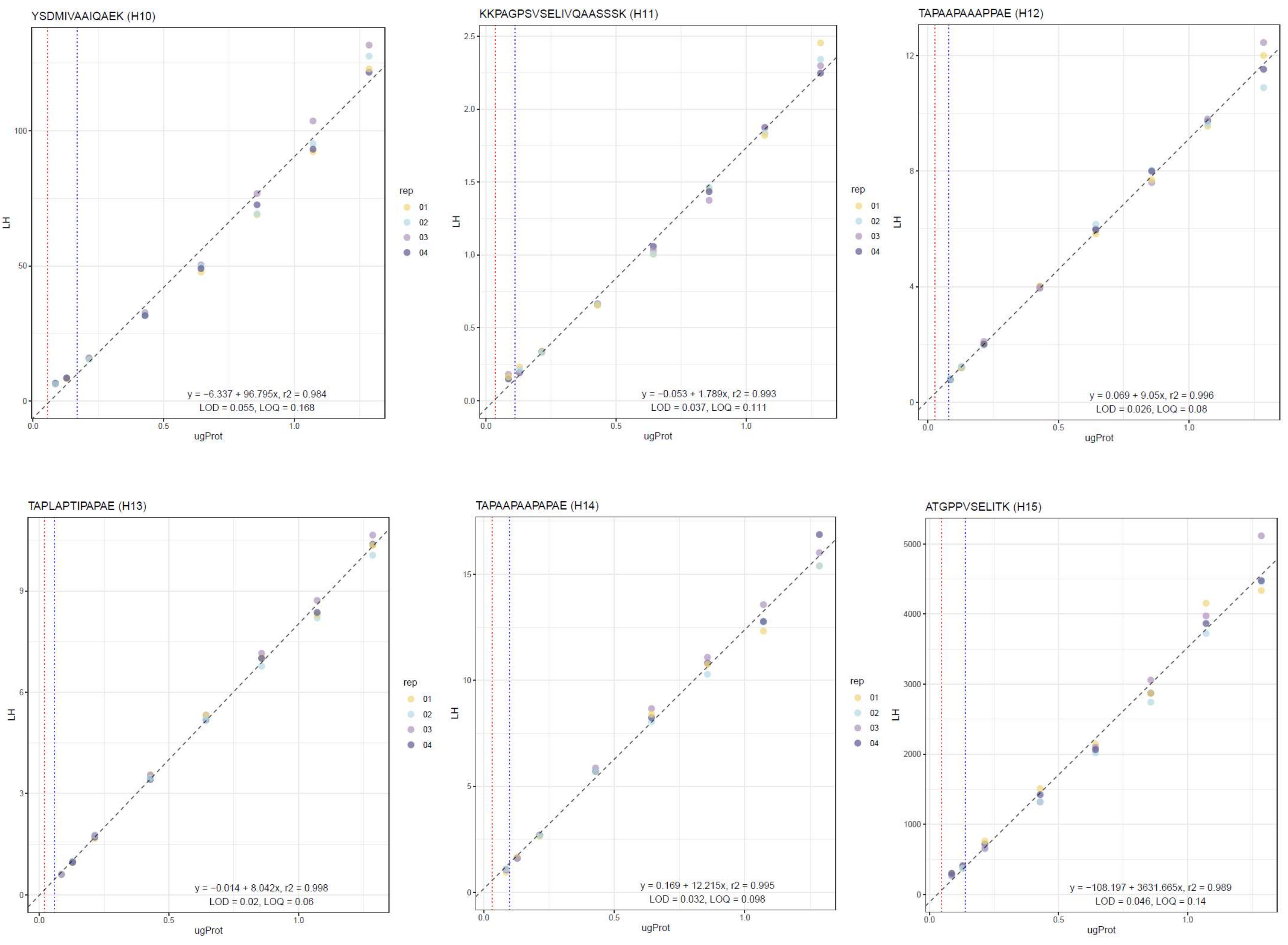

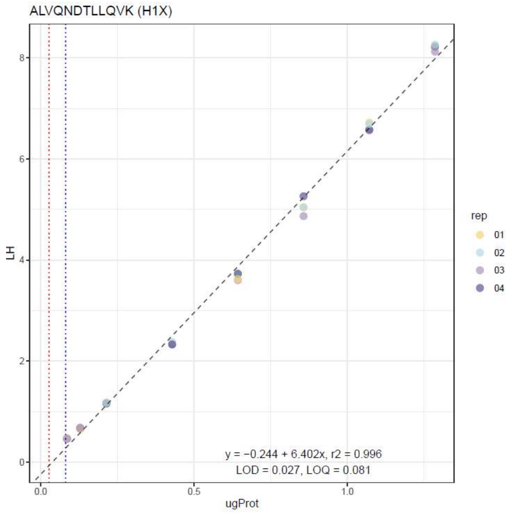

Figure S3. Calibration curves of the heavy peptides used for quantification of H1 subtypes. All the curves were obtained using four replicates of each data point. The equation shown was used to calculate the limits of detection (LOD) and quantification (LOQ), as described in materials and methods.

Figure S4

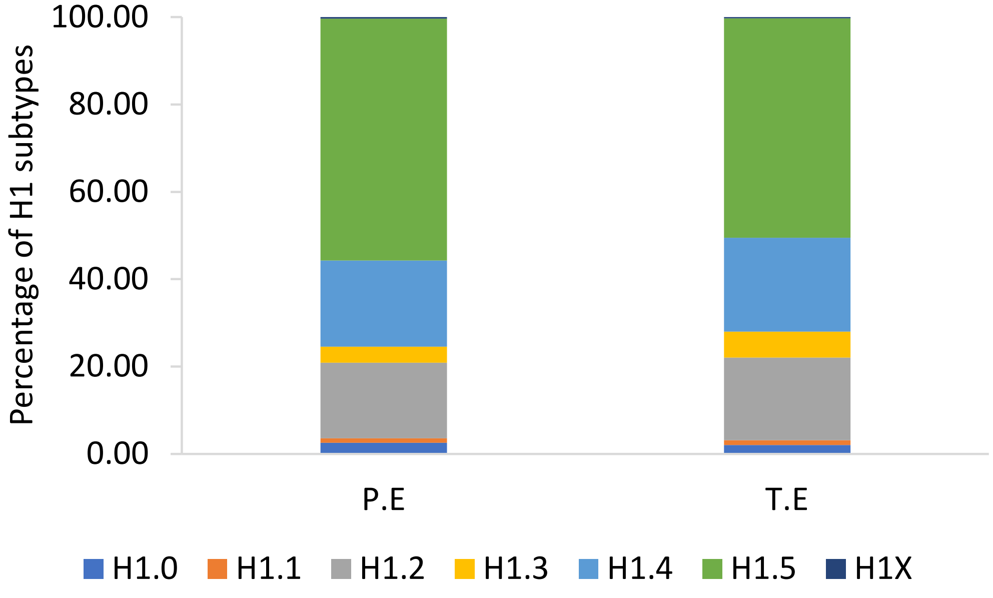

Figure S4. Proportions of H1 subtypes in the pool sample quantified from perchloric acid extracts (P.E) and total protein extracts (T.E).

Figure S5

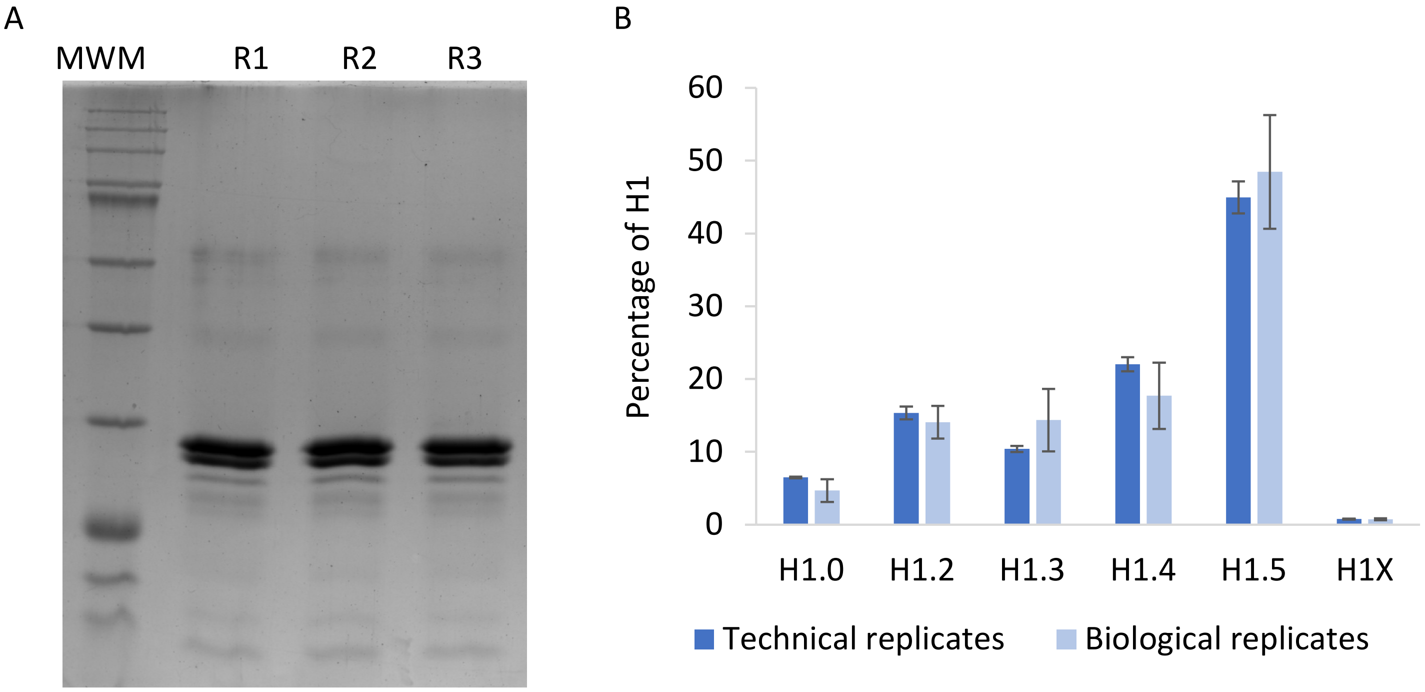

Figure S5. Comparison between biological and technical replicates. A.SDS-PAGE of the perchloric acid extractions of the three biological replicates. B. Percentages of the somatic H1 subtypes. Error bars correspond to the standard deviation of three technical or biological replicates as specified in the legend. MWM, molecular weight marker (NZYtech). R1, R2, and R3 are replicates 1, 2, and 3, respectively.

Figure S6

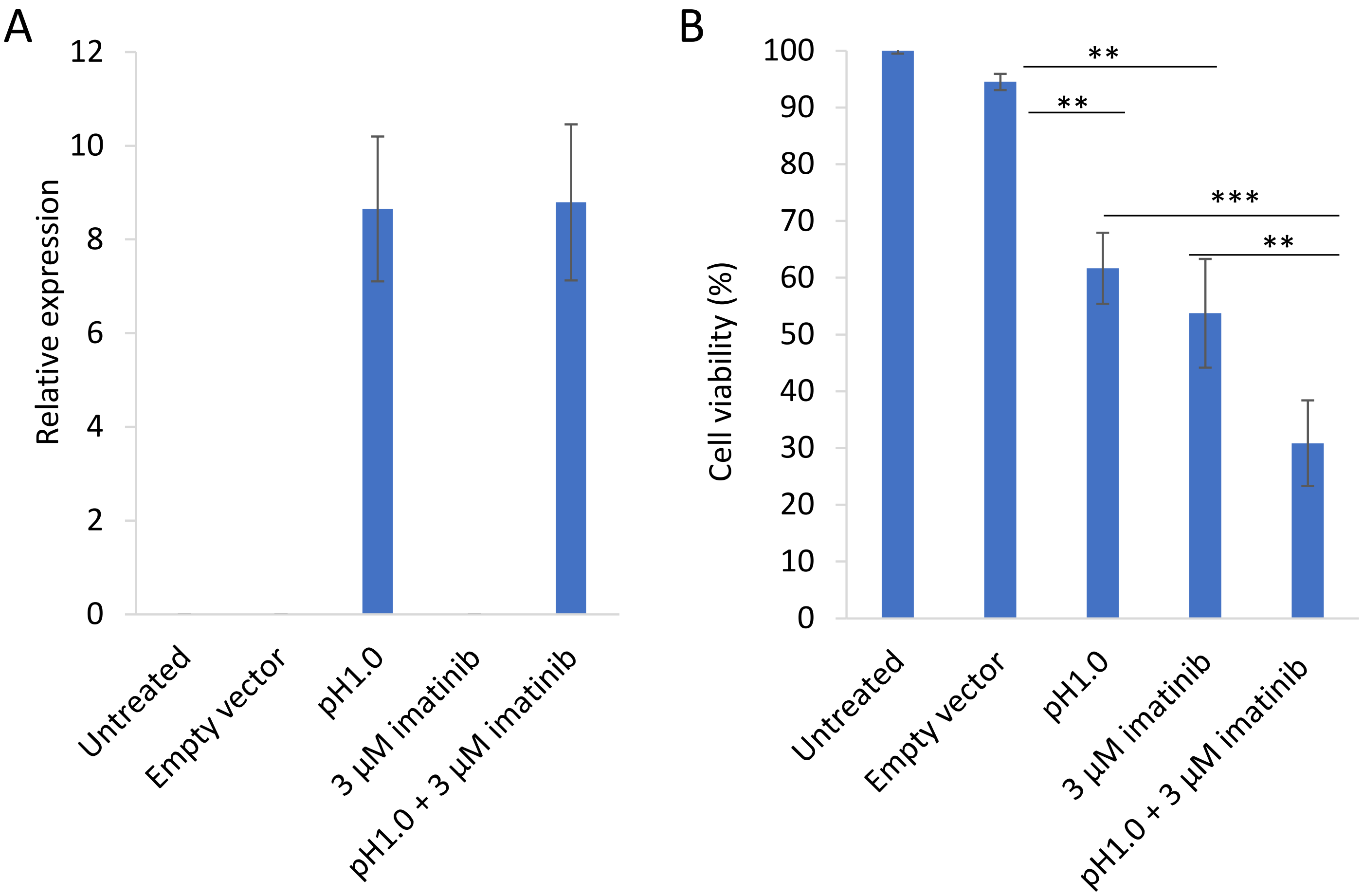

Figure S6. Exogenous expression of H1.0 in K562 cells. A. Relative expression of H1.0 to GAPDH. Cq values in the untreated K562 cells, K562 cells transfected with the empty vector, and those treated with 3 µM imatinib for 24h were similar to the control without reverse transcriptase(-RT), so the gene is considered silent. B. Cell viability expressed as a percentage of the untreated K562 cells. Error bars correspond to the standard deviation of three biological replicates. Differences in cell viability in two different conditions were analyzed using a two-tailed t-test. ** p-value < 0.01 and *** p-value < 0.001. Only the relevant comparisons are shown. pH1.0: plasmid containing the coding region of H1.0.
